# Supplementary material for: Heparin-based hydrogel scaffolding alters the transcriptomic profile and increases the chemoresistance of MDA-MB-231 triple-negative breast cancer cells
Source: Biomater Sci. 2020 Feb 13;8(10):2786–96. doi: 10.1039/c9bm01481k (PMC7497406; doi:10.1039/c9bm01481k)
Supplement: Supplementary file 2 [file BM-008-C9BM01481K-s002.zip › Supplementary File 4/HP-B hydrogel + EGF (s)vControl/Pathways/my_analysis.Gsea.1545201170987/HALLMARK_HEME_METABOLISM.html]

Details for gene set HALLMARK\_HEME\_METABOLISM[GSEA]

|  || Dataset | expr.class.cls#HYDEGF\_versus\_CONTROL.class.cls#HYDEGF\_versus\_CONTROL\_repos |
| Phenotype | class.cls#HYDEGF\_versus\_CONTROL\_repos |
| Upregulated in class | CONTROL |
| GeneSet | HALLMARK\_HEME\_METABOLISM |
| Enrichment Score (ES) | -0.35325733 |
| Normalized Enrichment Score (NES) | -1.6667097 |
| Nominal p-value | 0.0 |
| FDR q-value | 0.004740106 |
| FWER p-Value | 0.059 |
Table: GSEA Results Summary

  

Fig 1: Enrichment plot: HALLMARK\_HEME\_METABOLISM      
 Profile of the Running ES Score & Positions of GeneSet Members on the Rank Ordered List

  

| PROBE | DESCRIPTION (from dataset) | GENE SYMBOL | GENE\_TITLE | RANK IN GENE LIST | RANK METRIC SCORE | RUNNING ES | CORE ENRICHMENT || 1 | PQLC1 | na |  |  | 287 | 2.406 | 0.0017 | No |
| 2 | TNS1 | na |  |  | 368 | 2.281 | 0.0134 | No |
| 3 | AGPAT4 | na |  |  | 432 | 2.196 | 0.0254 | No |
| 4 | E2F2 | na |  |  | 687 | 1.916 | 0.0254 | No |
| 5 | SPTB | na |  |  | 722 | 1.896 | 0.0368 | No |
| 6 | ANK1 | na |  |  | 891 | 1.760 | 0.0402 | No |
| 7 | OSBP2 | na |  |  | 969 | 1.709 | 0.0481 | No |
| 8 | RANBP10 | na |  |  | 1266 | 1.530 | 0.0432 | No |
| 9 | SYNJ1 | na |  |  | 1332 | 1.501 | 0.0502 | No |
| 10 | ASNS | na |  |  | 1455 | 1.448 | 0.0539 | No |
| 11 | RNF123 | na |  |  | 1470 | 1.442 | 0.0632 | No |
| 12 | RBM5 | na |  |  | 1515 | 1.426 | 0.0708 | No |
| 13 | MOCOS | na |  |  | 1750 | 1.343 | 0.0679 | No |
| 14 | MFHAS1 | na |  |  | 1780 | 1.335 | 0.0757 | No |
| 15 | PSMD9 | na |  |  | 1799 | 1.328 | 0.0840 | No |
| 16 | PIGQ | na |  |  | 2197 | 1.212 | 0.0716 | No |
| 17 | MAP2K3 | na |  |  | 2199 | 1.211 | 0.0800 | No |
| 18 | FBXO9 | na |  |  | 2522 | 1.122 | 0.0709 | No |
| 19 | KHNYN | na |  |  | 2527 | 1.121 | 0.0785 | No |
| 20 | SLC22A4 | na |  |  | 2596 | 1.106 | 0.0826 | No |
| 21 | MARK3 | na |  |  | 2864 | 1.037 | 0.0758 | No |
| 22 | CTNS | na |  |  | 2895 | 1.028 | 0.0814 | No |
| 23 | EZH1 | na |  |  | 2960 | 1.014 | 0.0851 | No |
| 24 | CROCCP2 | na |  |  | 2973 | 1.011 | 0.0915 | No |
| 25 | UCP2 | na |  |  | 3188 | 0.963 | 0.0870 | No |
| 26 | ABCB6 | na |  |  | 3396 | 0.922 | 0.0825 | No |
| 27 | ATP6V0A1 | na |  |  | 3443 | 0.915 | 0.0865 | No |
| 28 | BMP2K | na |  |  | 3828 | 0.841 | 0.0722 | No |
| 29 | ALDH1L1 | na |  |  | 4460 | 0.739 | 0.0442 | No |
| 30 | HBQ1 | na |  |  | 4531 | 0.730 | 0.0456 | No |
| 31 | MPP1 | na |  |  | 5002 | 0.652 | 0.0254 | No |
| 32 | SIDT2 | na |  |  | 5090 | 0.640 | 0.0253 | No |
| 33 | ARHGEF12 | na |  |  | 5445 | 0.584 | 0.0108 | No |
| 34 | NR3C1 | na |  |  | 5797 | 0.533 | -0.0039 | No |
| 35 | HMBS | na |  |  | 5973 | 0.506 | -0.0096 | No |
| 36 | ARL2BP | na |  |  | 6077 | 0.492 | -0.0116 | No |
| 37 | FOXO3 | na |  |  | 6580 | 0.427 | -0.0350 | No |
| 38 | ABCG2 | na |  |  | 6734 | 0.405 | -0.0402 | No |
| 39 | PPP2R5B | na |  |  | 6949 | 0.379 | -0.0488 | No |
| 40 | RAD23A | na |  |  | 7249 | 0.337 | -0.0622 | No |
| 41 | SLC25A38 | na |  |  | 7291 | 0.331 | -0.0620 | No |
| 42 | MARCH2 | na |  |  | 7585 | 0.294 | -0.0754 | No |
| 43 | SLC30A1 | na |  |  | 7822 | 0.260 | -0.0860 | No |
| 44 | DCAF11 | na |  |  | 7825 | 0.260 | -0.0842 | No |
| 45 | TFRC | na |  |  | 7904 | 0.255 | -0.0866 | No |
| 46 | UROS | na |  |  | 7943 | 0.250 | -0.0868 | No |
| 47 | XK | na |  |  | 8036 | 0.239 | -0.0900 | No |
| 48 | TSPAN5 | na |  |  | 8070 | 0.233 | -0.0901 | No |
| 49 | SEC14L1 | na |  |  | 8284 | 0.210 | -0.0998 | No |
| 50 | TRAK2 | na |  |  | 8297 | 0.210 | -0.0990 | No |
| 51 | FBXO34 | na |  |  | 8340 | 0.203 | -0.0998 | No |
| 52 | SLC7A11 | na |  |  | 8638 | 0.165 | -0.1142 | No |
| 53 | SLC10A3 | na |  |  | 8739 | 0.159 | -0.1184 | No |
| 54 | TSPO2 | na |  |  | 8768 | 0.155 | -0.1188 | No |
| 55 | SLC6A9 | na |  |  | 8802 | 0.150 | -0.1195 | No |
| 56 | CCND3 | na |  |  | 8864 | 0.140 | -0.1217 | No |
| 57 | GCLC | na |  |  | 8925 | 0.131 | -0.1239 | No |
| 58 | GAPVD1 | na |  |  | 9143 | 0.110 | -0.1346 | No |
| 59 | PPOX | na |  |  | 9160 | 0.108 | -0.1346 | No |
| 60 | KAT2B | na |  |  | 9997 | 0.022 | -0.1784 | No |
| 61 | PGLS | na |  |  | 10085 | 0.008 | -0.1829 | No |
| 62 | RAP1GAP | na |  |  | 10310 | -0.024 | -0.1945 | No |
| 63 | ADIPOR1 | na |  |  | 10332 | -0.026 | -0.1955 | No |
| 64 | UBAC1 | na |  |  | 10364 | -0.031 | -0.1969 | No |
| 65 | IGSF3 | na |  |  | 10576 | -0.048 | -0.2076 | No |
| 66 | GCLM | na |  |  | 10721 | -0.067 | -0.2147 | No |
| 67 | RBM38 | na |  |  | 10726 | -0.068 | -0.2145 | No |
| 68 | NEK7 | na |  |  | 10732 | -0.069 | -0.2142 | No |
| 69 | CLIC2 | na |  |  | 10962 | -0.101 | -0.2256 | No |
| 70 | BCAM | na |  |  | 11029 | -0.111 | -0.2283 | No |
| 71 | NARF | na |  |  | 11063 | -0.116 | -0.2292 | No |
| 72 | CPOX | na |  |  | 11194 | -0.122 | -0.2352 | No |
| 73 | TCEA1 | na |  |  | 11214 | -0.125 | -0.2353 | No |
| 74 | HAGH | na |  |  | 11365 | -0.142 | -0.2422 | No |
| 75 | FOXJ2 | na |  |  | 11447 | -0.153 | -0.2454 | No |
| 76 | RCL1 | na |  |  | 11478 | -0.160 | -0.2458 | No |
| 77 | ADD1 | na |  |  | 11543 | -0.167 | -0.2480 | No |
| 78 | RHD | na |  |  | 11907 | -0.214 | -0.2656 | No |
| 79 | NNT | na |  |  | 12167 | -0.239 | -0.2776 | No |
| 80 | TFDP2 | na |  |  | 12198 | -0.243 | -0.2774 | No |
| 81 | EPB41 | na |  |  | 12209 | -0.245 | -0.2763 | No |
| 82 | TNRC6B | na |  |  | 12250 | -0.252 | -0.2766 | No |
| 83 | H1F0 | na |  |  | 12323 | -0.265 | -0.2785 | No |
| 84 | MARCH8 | na |  |  | 12631 | -0.302 | -0.2926 | No |
| 85 | LMO2 | na |  |  | 12668 | -0.308 | -0.2923 | No |
| 86 | CTSE | na |  |  | 13010 | -0.343 | -0.3078 | No |
| 87 | ATG4A | na |  |  | 13037 | -0.346 | -0.3068 | No |
| 88 | SLC2A1 | na |  |  | 13117 | -0.356 | -0.3085 | No |
| 89 | FN3K | na |  |  | 13127 | -0.358 | -0.3064 | No |
| 90 | PC | na |  |  | 13181 | -0.366 | -0.3067 | No |
| 91 | FECH | na |  |  | 13413 | -0.405 | -0.3160 | No |
| 92 | HDGF | na |  |  | 13545 | -0.420 | -0.3199 | No |
| 93 | GMPS | na |  |  | 13591 | -0.423 | -0.3194 | No |
| 94 | NFE2L1 | na |  |  | 13688 | -0.440 | -0.3213 | No |
| 95 | CAST | na |  |  | 13782 | -0.455 | -0.3231 | No |
| 96 | TMEM9B | na |  |  | 13801 | -0.458 | -0.3208 | No |
| 97 | BTG2 | na |  |  | 14115 | -0.493 | -0.3338 | No |
| 98 | SLC6A8 | na |  |  | 14163 | -0.502 | -0.3328 | No |
| 99 | NFE2 | na |  |  | 14182 | -0.504 | -0.3302 | No |
| 100 | USP15 | na |  |  | 14247 | -0.513 | -0.3300 | No |
| 101 | SMOX | na |  |  | 14278 | -0.516 | -0.3280 | No |
| 102 | GLRX5 | na |  |  | 14353 | -0.529 | -0.3282 | No |
| 103 | BTRC | na |  |  | 14450 | -0.549 | -0.3294 | No |
| 104 | DAAM1 | na |  |  | 14563 | -0.567 | -0.3314 | No |
| 105 | XPO7 | na |  |  | 14642 | -0.579 | -0.3314 | No |
| 106 | MINPP1 | na |  |  | 14880 | -0.616 | -0.3396 | No |
| 107 | ALAD | na |  |  | 15047 | -0.642 | -0.3438 | No |
| 108 | EPOR | na |  |  | 15162 | -0.662 | -0.3452 | No |
| 109 | AQP3 | na |  |  | 15228 | -0.674 | -0.3439 | No |
| 110 | CDR2 | na |  |  | 15270 | -0.683 | -0.3413 | No |
| 111 | SLC25A37 | na |  |  | 15435 | -0.707 | -0.3450 | No |
| 112 | ENDOD1 | na |  |  | 15520 | -0.725 | -0.3444 | No |
| 113 | ISCA1 | na |  |  | 15576 | -0.738 | -0.3421 | No |
| 114 | MBOAT2 | na |  |  | 15789 | -0.782 | -0.3478 | Yes |
| 115 | BSG | na |  |  | 15875 | -0.796 | -0.3467 | Yes |
| 116 | ELL2 | na |  |  | 15882 | -0.798 | -0.3415 | Yes |
| 117 | RNF19A | na |  |  | 15886 | -0.799 | -0.3361 | Yes |
| 118 | TMCC2 | na |  |  | 15906 | -0.803 | -0.3315 | Yes |
| 119 | GDE1 | na |  |  | 15928 | -0.807 | -0.3270 | Yes |
| 120 | OPTN | na |  |  | 15986 | -0.816 | -0.3243 | Yes |
| 121 | FBXO7 | na |  |  | 16227 | -0.880 | -0.3308 | Yes |
| 122 | SLC11A2 | na |  |  | 16327 | -0.906 | -0.3296 | Yes |
| 123 | ERMAP | na |  |  | 16415 | -0.926 | -0.3278 | Yes |
| 124 | DCAF10 | na |  |  | 16439 | -0.931 | -0.3225 | Yes |
| 125 | LRP10 | na |  |  | 16479 | -0.941 | -0.3180 | Yes |
| 126 | DCUN1D1 | na |  |  | 16531 | -0.952 | -0.3140 | Yes |
| 127 | HTRA2 | na |  |  | 16814 | -1.031 | -0.3216 | Yes |
| 128 | HEBP1 | na |  |  | 17118 | -1.117 | -0.3298 | Yes |
| 129 | CDC27 | na |  |  | 17136 | -1.124 | -0.3228 | Yes |
| 130 | BACH1 | na |  |  | 17150 | -1.128 | -0.3157 | Yes |
| 131 | PICALM | na |  |  | 17171 | -1.134 | -0.3088 | Yes |
| 132 | NUDT4 | na |  |  | 17172 | -1.134 | -0.3009 | Yes |
| 133 | C3 | na |  |  | 17200 | -1.144 | -0.2944 | Yes |
| 134 | HTATIP2 | na |  |  | 17236 | -1.155 | -0.2882 | Yes |
| 135 | MKRN1 | na |  |  | 17260 | -1.165 | -0.2812 | Yes |
| 136 | EIF2AK1 | na |  |  | 17298 | -1.181 | -0.2750 | Yes |
| 137 | PDZK1IP1 | na |  |  | 17416 | -1.221 | -0.2726 | Yes |
| 138 | BLVRB | na |  |  | 17508 | -1.262 | -0.2686 | Yes |
| 139 | NCOA4 | na |  |  | 17593 | -1.292 | -0.2640 | Yes |
| 140 | HIST1H4C | na |  |  | 17641 | -1.310 | -0.2573 | Yes |
| 141 | UROD | na |  |  | 17690 | -1.327 | -0.2506 | Yes |
| 142 | RIOK3 | na |  |  | 17725 | -1.347 | -0.2430 | Yes |
| 143 | P4HA2 | na |  |  | 17913 | -1.447 | -0.2428 | Yes |
| 144 | CLCN3 | na |  |  | 17982 | -1.478 | -0.2360 | Yes |
| 145 | LPIN2 | na |  |  | 18001 | -1.486 | -0.2266 | Yes |
| 146 | PRDX2 | na |  |  | 18021 | -1.496 | -0.2172 | Yes |
| 147 | VEZF1 | na |  |  | 18054 | -1.513 | -0.2083 | Yes |
| 148 | BLVRA | na |  |  | 18074 | -1.527 | -0.1987 | Yes |
| 149 | ALDH6A1 | na |  |  | 18145 | -1.576 | -0.1914 | Yes |
| 150 | TOP1 | na |  |  | 18213 | -1.609 | -0.1837 | Yes |
| 151 | CIR1 | na |  |  | 18249 | -1.637 | -0.1742 | Yes |
| 152 | MOSPD1 | na |  |  | 18287 | -1.666 | -0.1645 | Yes |
| 153 | SELENBP1 | na |  |  | 18289 | -1.668 | -0.1529 | Yes |
| 154 | CCDC28A | na |  |  | 18346 | -1.709 | -0.1440 | Yes |
| 155 | MGST3 | na |  |  | 18477 | -1.823 | -0.1381 | Yes |
| 156 | CTSB | na |  |  | 18524 | -1.856 | -0.1276 | Yes |
| 157 | FAM46C | na |  |  | 18621 | -1.949 | -0.1190 | Yes |
| 158 | CAT | na |  |  | 18668 | -2.014 | -0.1074 | Yes |
| 159 | YPEL5 | na |  |  | 18775 | -2.152 | -0.0980 | Yes |
| 160 | LAMP2 | na |  |  | 18797 | -2.190 | -0.0838 | Yes |
| 161 | SDCBP | na |  |  | 18811 | -2.209 | -0.0691 | Yes |
| 162 | KLF3 | na |  |  | 18891 | -2.340 | -0.0570 | Yes |
| 163 | SLC30A10 | na |  |  | 18917 | -2.388 | -0.0416 | Yes |
| 164 | BNIP3L | na |  |  | 18957 | -2.475 | -0.0264 | Yes |
| 165 | MXI1 | na |  |  | 18988 | -2.556 | -0.0102 | Yes |
| 166 | BPGM | na |  |  | 19126 | -3.042 | 0.0038 | Yes |
Table: GSEA details [plain text format]

  

Fig 2: HALLMARK\_HEME\_METABOLISM      
 Blue-Pink O' Gram in the Space of the Analyzed GeneSet

  

Fig 3: HALLMARK\_HEME\_METABOLISM: Random ES distribution      
 Gene set null distribution of ES for **HALLMARK\_HEME\_METABOLISM**
